# Is Malaysia Ready for Human Gene Editing: A Regulatory, Biosafety and Biosecurity Perspective

**DOI:** 10.3389/fbioe.2021.649203

**Published:** 2021-03-11

**Authors:** V. Kalidasan, Kumitaa Theva Das

**Affiliations:** Infectomics Cluster, Advanced Medical and Dental Institute, Universiti Sains Malaysia, Kepala Batas, Malaysia

**Keywords:** Malaysia, gene editing, CRISPR, gene therapy, regulation, biosafety, biosecurity

## Abstract

Gene editing platforms have revolutionized the field of genetics with a direct impact on the public health system. Although there are apparent benefits, it is often accompanied by public debates over its uncertainties and risks. In the Malaysian context, modern biotechnology has raised questions about how to best govern gene editing in regulations, biosafety, and biosecurity. Even though standards and guidelines on stem cell and cell-based therapies have been developed, there are no appropriate legal frameworks available for gene editing yet. Nevertheless, biosafety regulations were established to balance promoting biotechnology and protecting against their potential environmental and human health risks. There is also a need to address the potential of genetically modified organisms (GMOs) as bioweapons. Numerous frameworks from several international organizations may provide valuable input in formulating documents on gene editing. By establishing comprehensive guidelines, legal policies, and standards to tackle the challenges and risks associated with gene editing, Malaysia can successfully apply this modern technology in this country.

## Introduction

Population health is widely recognized as a critical indicator of economic growth in a country (Lange and Vollmer, 2017). Malaysia’s growth was substantial in 2019, whereby the gross domestic product (GDP) was RM1.51 trillion, and their gross national income (GNI) per capita increased from RM 43,307 to RM 45,131 that same year. Overall, the economy expanded by 4.3% in 2019, compared to 4.8% in the preceding year (Department of Statistics Malaysia, 2020). Under such circumstances, it is essential to ensure that health resources benefit the population, thereby enabling citizens to strengthen economic performance. Although the burden of disease in Malaysia is manageable by public and private healthcare systems (Quek, 2009; Thomas et al., 2011), the demand for treatment and disease prevention is still a significant challenge.

With the emergence of new medical technologies ranging from smart inhalers, robotic surgery, wireless brain sensors, 3-D printing, artificial organs, health wearables, virtual reality to precision medicine, and gene editing, Malaysia could have a tremendous breakthrough (Ellis, 2019). Precision medicine (also known as personalized medicine) is driven by genome sequencing technologies and data science, allowing clinicians to tailor treatments individually based on genes, environment, and lifestyle factors (Academy of Sciences Malaysia, 2009; Jamal, 2017). Notably, precision medicine is already practiced in Malaysia with a high success rate, such as treating cancer through a tumor profiling approach that can identify various anti-cancer-therapies (Murugesan, 2019).

Another crucial advancement that has gained much attention worldwide is gene editing technology. Malaysia has made progress in medical genetics, with some researchers using genome editing to delete, insert, or modify DNA sequences to correct a particular disease (Hamid, 2018; Nithya et al., 2019). Despite its potential, there is a high demand for an ethics panel to develop guidelines for human genome editing in Malaysia, especially for germline editing (Fong, 2019). In such circumstances, governing the use of genome editing to improve healthcare, balancing potential benefits with unintended risks, and integrating societal values in the therapeutic application and decision-making is of utmost importance. Thus, this review aims to debate the regulatory, biosafety, and biosecurity aspects of gene editing in Malaysia.

## Genome Editing: Basic Sciences and its Therapeutic Applications

**FIGURE 1:**
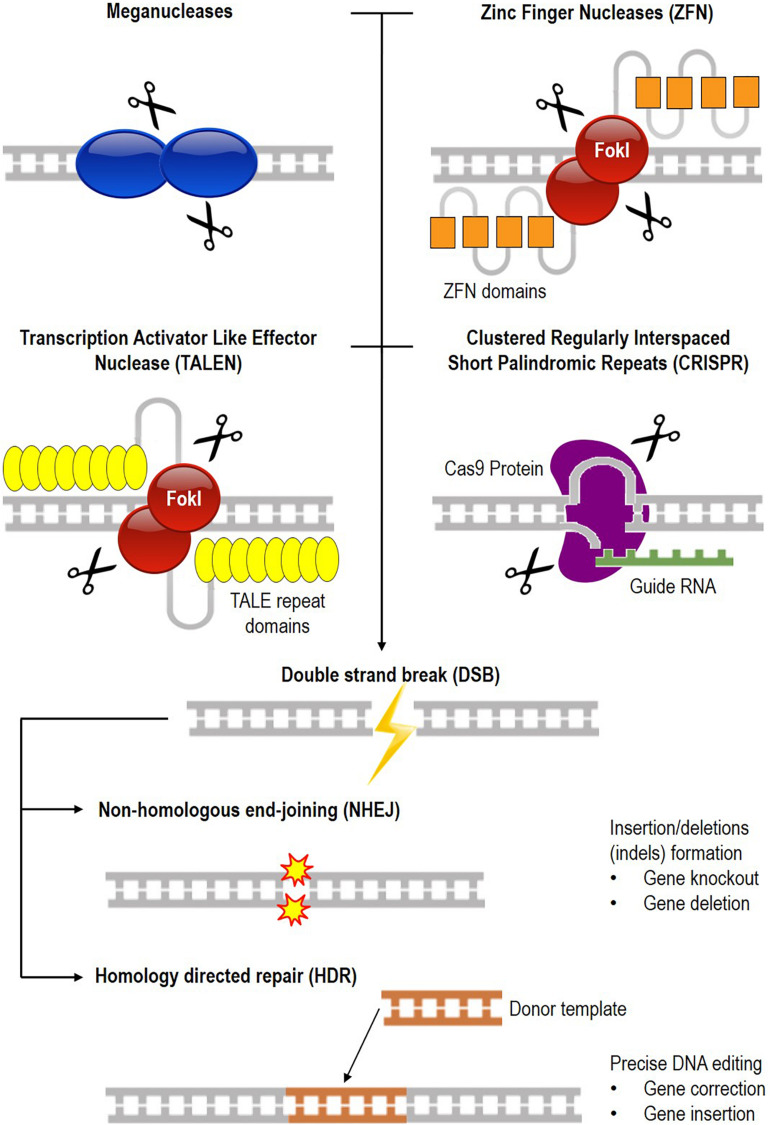
Common DNA targeting platform for genome editing. There are currently four different nucleases available for gene editing which are meganuclease, Zinc Finger Nuclease (ZFN), Transcription Activator Like Effector Nuclease (TALEN), and Clustered Regularly Interspaced Short Palindromic Repeats (CRISPR). DNA is cleaved (scissors symbol), resulting in a double-stranded break (DSB) that is repaired by either non-homologous end-joining (NHEJ) or homology-directed repair (HDR). NHEJ results in the formation of insertions or deletions (indels) for gene knock-out or deletion, while in HDR, a donor DNA repairs the broken ends of the chromosome for gene correction or insertion.

Gene editing involves creating a specific double-stranded break (DSB) in the genome, followed by cellular repair mechanisms (Porteus, 2015; Mandip and Steer Clifford, 2019), either through non-homologous end-joining (NHEJ) where indels are created at the break site or homology-directed repair (HDR) where a specific nucleotide change takes place in the genome with the help of a donor sequence. Currently, four leading platforms exist for genome editing, namely engineered meganuclease, Zinc Finger Nuclease (ZFN), Transcription Activator Like Effector Nuclease (TALEN), and Clustered Regularly Interspaced Short Palindromic Repeats (CRISPR) (Ben-David, 2013; Ramalingam et al., 2013; Kim, 2016). The various generations of nucleases used for genome editing and their DNA repair mechanisms are illustrated in Figure 1, and a comparison of the different programmable nuclease platforms is shown in Table 1.

**TABLE 1:** Systematic comparison of meganuclease, Zinc Finger Nuclease (ZFN), Transcription Activator Like Effector Nuclease (TALEN), and Clustered Regularly Interspaced Short Palindromic Repeats (CRISPR) genome editing platforms.

**Features**	**Meganuclease**	**ZFN**	**TALEN**	**CRISPR/Cas9**
Source	Organellar DNA, bacteria, phage	Bacteria, eukaryotes	Bacteria (*Xanthomonas* sp.)	Bacteria (*S. pyogenes*)
Polymeric state	Dimers (two identical subunits)	Dimers (two FokI domains)	Dimers (two FokI domains)	Monomer (only sgRNA-Cas9 complex)
Type of recognition	Protein-DNA	Protein-DNA	Protein-DNA	RNA-DNA
Recognition site	Between 18 and 44 bp	Between 18 and 36 bp	Between 24 and 40 bp	Between 17 and 23 bp
DSB pattern	Staggered (3′ overhang)	Staggered (5′ overhang)	Staggered (heterogenous overhang)	Staggered (5′ overhang, Cpf1 system); blunt (SpCas9)
Specificity	High	Low to moderate	Moderate	Low to moderate
Ease of design and engineering	Difficult	Difficult	Moderate	Easy
Immunogenicity	Unknown	Low	Unknown	Unknown
*Ex vivo* delivery	Easy using electroporation and viral vector	Easy using electroporation, viral vector and lipofection	Easy using electroporation, viral vector and lipofection	Easy using electroporation, viral vector and lipofection
*In vivo* delivery	Easy to difficult (depending on size of nuclease)	Easy to difficult (depending on size of nuclease)	Difficult (large size of TALEN)	Moderate (*S. pyogenes* is large)
Multiplexing	Low	Low	Moderate to high	High
Cost (USD)	4,000–5,000	5–10,000	Less than 1,000	Less than 100
Success rate	Low	Low (∼24%)	High (>99%)	High (∼90%)
Targeting constraints	Targeting novel sequencing	Targeting non-G-rich sequence	5′ targeted base must be a T for each TALEN monomer	Targeted sequence must precede a PAM sequence
Advantages	Possible to edit various types of genome editing (knockout, reporter, specific alleles)	Designed to target any DNA sequence; targeting of biallelic genes	Designed to target any DNA sequence; targeting of biallelic genes	Targeting of biallelic genes and multiplexing
Disadvantages	Lacks DNA-binding domains; inefficient for inadequate knowledge on designing construct; time-consuming	Binding capacity of ZFN depends on neighboring ZFs; decreased specificity can lead to off-target cleavage	Cloning of TALE repeats is troublesome and error prone	Target sites limited for PAM motif; higher chance of off-target cleavage

With the ease of genome editing, the pace of progress has increased exponentially. Many organisms have already been genetically modified, such as mice, rats, monkeys, pigs, cows, rabbits, frogs, zebrafish, fruit flies, worms, yeast, and bacteria (Gersbach, 2014). These species have contributed to the studies of genetics, genomics, gene function, and disease modeling. The most significant benefit of genome editing is undoubtedly applying these technologies to improve human health through gene therapy (Cox et al., 2015; Mandip and Steer Clifford, 2019). Numerous human diseases have already been targeted for gene therapy and have moved into preclinical phases such as viral infections, T-cell immunotherapy, hematological disorder, neuromuscular disorders, skin disorders, respiratory disorders, and many others.

In general, gene therapy can be broadly categorized into somatic and germline therapy. Somatic gene therapy involves changes to cells (i.e., bone marrow, blood, and skin) that are limited to the treated individual and would not be inherited by future generations (Smith, 2003). Broadly, alteration on somatic cells can be done either by *in vivo* modification targeting specific tissues with local delivery into the body or *ex vivo* modification targeting cells outside the body, followed by reinfusion of the edited cells. In terms of therapeutic delivery of genetic material (transgene), two approaches can be used: (i) viral delivery using a retrovirus, adenovirus, and adeno-associated viruses (AAV), or (ii) non-viral delivery using liposome, electroporation, tissue injection, and particle bombardment.

Before performing somatic cell genome editing, a few points should be considered (National Academies of Sciences Engineering and Medicine, 2017c), including which cells or tissue(s) are modified, where the editing takes place (*in vivo* or *ex vivo*), specific goal(s) of the modification (treatment or prevention of disease or introduction of new traits), and the precise nature of the modification (changing disease-causing mutation, disruption or overexpression of an endogenous gene, or addition of a novel function). Notably, several additional features must be considered with both *in vivo* and *ex vivo* editing, such as the ability to isolate the relevant cell type (i.e., *ex vivo*), the ability to control biodistribution of the genome-editing tool (i.e., *in vivo*), the ability to limit immune response to delivery vectors that could lead to rapid and complete clearance of cells that have received the editing complex, and the ability to edit the genome in non-dividing cells (i.e., dividing cells such stem cells versus non-dividing cells such as neurons). Regardless of the application, each strategy needs to be evaluated in terms of safety, efficacy, risk, cost, and feasibility.

On the other hand, germline gene therapy involves modifying genes that will be passed to the next generation, thus not being widely attempted in humans. Germline therapy must be performed during the early stages of development on egg cells, embryonic stem cells, sperm cells using pronuclear microinjection or nuclear transfer (Smith, 2003). Liang et al. (2015) who published the first report of human embryo genetic engineering utilized tripronuclear (3PN) zygotes and edited a portion of the human β-globin gene using CRISPR/Cas9. Since the 3PN zygote would develop naturally into an embryo but does not result in birth (non-viable human zygotes), that embryo was used to avoid ethical concerns. The findings showed several off-target mutations resulting in mosaic embryos, highlighting the need for further investigation before clinical application. Another researcher from China, He Jiankui, performed germline gene therapy on twins babies Lulu and Nana (Ryder, 2018) where he injected the embryos with CRISPR/Cas9 to knock out CCR5 co-receptor to prevent HIV binding. Unfortunately, his findings revealed that only Nana would be resistant to HIV (the edits removed both copies of her CCR5 gene), while Lulu would still be susceptible to infection (she still had one functional copy of CCR5) (Cyranoski, 2018).

It is crucial to ensure that only embryos with correctly targeted alleles would be returned to the uterus to complete pregnancy (National Academies of Sciences Engineering and Medicine, 2017a) as some of the cells would not have the desired edits (mosaicism), and there may be unwanted effects of the removal of disease-causing variant on the human gene pool. Alternative routes should also be considered over heritable edits (i.e., using edited sperms to fertilize donor eggs) as it is inconclusive whether germline editing can be performed safely. All these factors must be evaluated carefully based on scientific and ethical grounds before considering germline therapy.

### Debate 1: What Are the Risks and Benefits of Modifying Human DNA? What Are the Arising Controversies of Gene Editing?

Jesse Gelsinger’s tragic death during his clinical trials turned gene therapy into a significant debate (Sibbald, 2001; Gelsinger, 2016). The 18-year-old American had a condition called ornithine transcarbamylase deficiency (OTC), where he lacked a functional enzyme that breaks down ammonia, and becomes toxic in higher concentrations. On 13th September 1999, he received an adenoviral vector injection (3.8 × 10^13^ particles) to introduce a normal gene for the enzyme directly into his liver (Savulescu, 2001). Unfortunately, he experienced a severe immune reaction to the vector and died 4 days after receiving the treatment.

His death highlighted a few ethical and legal issues (Savulescu, 2001; Sibbald, 2001). Firstly, he was not informed about the preclinical evidence of patients with dangerous side effects from the therapy or that three monkeys had died of a clotting disorder and severe liver inflammation after being injected. Secondly, the research team was careless, negligent, and reckless as they failed to evaluate Jesse’s condition adequately. Thirdly, prolonged storage of the vector for 25 months led researchers to underestimate its potency. Fourthly, there was a conflict of interest between the researchers and a private sector biotechnology collaborator in the project that prevented reporting any adverse effect to the Food and Drug Administration (FDA). Consequently, the U.S. Department of Justice directed all guilty parties to pay a sum of fines (Couzin and Kaiser, 2005). The court declared that a toxic reaction in humans should have halted the trial as early as possible, and the investigators misrepresented the clinical findings to the study’s regulators.

Moving forward, the First International Summit on Human Gene Editing (2015) recommended that all research must be subjected to appropriate legal and ethical rules and oversight (National Academies of Sciences Engineering and Medicine, 2015) and “rigorously evaluated existing and evolving regulatory frameworks for gene therapy clinical trials.” As of November 2017, 2597 trials were approved and undertaken in 38 countries, with most gene therapy clinical trials addressing cancer (i.e., gynecological, nervous, gastrointestinal, genitourinary, skin, lung, hematological), and inherited monogenic diseases (i.e., primary immunodeficiency disorders, cystic fibrosis) (Ginn et al., 2018). These gene therapy trials offered clear proof-of-concept, demonstrating safety, and emphasized critical issues for therapy advancement.

However, somatic modification could exert conflict of interest, particularly in behavioral genetics, physical traits, and sports science. Low levels of monoamine oxidase A (MAOA) have been reported among people who experienced maltreatment during childhood, resulting in violent behavior and increased crime rate as they age (Polcz and Lewis, 2016). In such a phenomenon, should gene therapy be initiated to lower the risk of violent outbursts? Should these offenders be regarded as lesser criminals due to their genetic predisposition? Another speculative issue on gene manipulation is gene doping among athletes to increase their performance, maximize bodily function, and alter muscle endurance (Battery et al., 2011). Considering gene editing would most likely not be detected during testing, the World Anti-Doping Agency (WADA) banned it in 2003. It is crucial to draw the line between therapeutic uses and gene editing enhancement (Cwik, 2019). The latter poses major ethical, societal, and regulatory issues that need to be acknowledged before allowing genetic enhancement to become a reality.

In terms of germline gene therapy, He Jiankui’s experiment caused much controversy in biomedical research (Normile, 2018; Ryder, 2018). The announcement of He’s heritable genome editing during the Second International Summit of Human Genome Editing (2018) caused scrutiny on inadequate oversight and transparency, lack of parental informed consent, the existence of alternative care for preventing infection, the likelihood that gene editing will cause other medical problems, and the source of research funding (National Academies of Sciences Engineering and Medicine, 2019). The scientific community believed that the risks and benefits of germline editing were unclear to allow it to proceed and called for a moratorium until there was broad consensus on the clinical use of genome editing, and an extensive regulatory framework, ethical framework, religious viewpoint, public and societal engagement prior to this technology moving forward (Porteus and Dann, 2015).

In general, germline genome editing’s ethical issues can be classified into those arising from its potential failure and success (Ormond et al., 2017; Coller, 2019). Firstly, the potential harm is perceived as a risk that does not outweigh the potential benefits. In germline editing, the unintended consequences are not well understood. In such circumstances, adopting national and international policies (i.e., legislation, regulation, and professional guidance), document enforcement (i.e., legally binding or self-compliances), and oversight mechanisms (i.e., licensing) would be the standard framework to addressing germline genome editing. Secondly, if the technology works as intended, the individual, family, and society would be largely impacted. The technique affects the person’s future, whose genes are altered without their consent. Even though parents hold the decision-making capacity, there may be individuals who did not wish to remove their medical conditions and disagree with the decision made by their parents. On the other hand, parents may believe that such interventions are intended to reduce potential harm to the child. In this scenario, there is an evident conflict between informed consent and non-maleficence.

There are significant concerns about eugenics, social justice, and equal access to therapy (Coller, 2019). Eugenics is a concept that retains positive traits and removes negative characteristics. In such a context, germline modification may result in the loss of genetic diversity in the future generation and create children with the best traits (designer babies). Many consider this as ‘playing God,’ while some believe that it is merely altering genes rather than selecting against individuals. These issues raise an argument related to genetic enhancement where the manipulation for physical and mental abilities, and knowledge may most likely result in professional success. Since human germline therapy would probably only be affordable to people from a specific socioeconomic class, the central dilemma is that individuals who have the resources would obtain unfair success.

Despite these ethical and social concerns, the National Academies of Sciences, Engineering, and Medicine (NASEM) recommended that clinical trials on heritable human genome editing proceed for limited purposes, under these following conditions (National Academies of Sciences Engineering and Medicine, 2017a): (i) the absence of reasonable alternatives, (ii) limited to editing genes that have been demonstrated to strongly cause or to predispose to a disease, (iii) restricted to converting genes to versions that are prevalent in the population and are known to be associated with ordinary health with little or no evidence of adverse effects, (iv) the availability of credible preclinical and/or clinical data on risks and potential health benefits of the procedures, (v) ongoing, rigorous oversight during clinical trials on the effects of the procedure on the health and safety of the research participants, (vi) comprehensive plans for long-term, multigenerational follow-up that respects personal rights, (vii) maximum transparency consistent with patient privacy, (viii) continued assessment of health and societal benefits and risks, with broad ongoing participation and input by the public, and (ix) reliable oversight mechanisms to prevent extension to uses other than preventing a severe disease. In short, the development and application of somatic and germline therapy should consider conducting careful scientific research to build an evidence-based study, evaluating ethical, legal, and social issues (ELSI), and conducting meaningful stakeholder engagement, education, and dialogue (SEED) (Howard et al., 2018). The adapted questions that should be discussed for each of the mentioned aspects are tabulated in Table 2.

**TABLE 2:** Example of questions for conducting careful scientific research, ethical, legal, and social issues (ELSI) research, and meaningful stakeholder engagement, education, and dialogue (SEED) in context of gene editing.

**Aspects**	**Example of questions**
**Building a scientific evidence base for gene editing**	
Carry out ongoing responsible scientific research to create a solid foundation of facts, especially with regard to risks and benefits	• Is the current standards and practices of sharing academic and commercial research results, in particular with regard to risks and benefits, adequate for the current and future gene editing field? • Should there be a common framework developed for tracking (systematically) all forms of basic and (pre) clinical research? • If so, which kind of work does it take to adhere to this? All research, or just work done in human cells? • Who should/will be taking responsibility for tracking or reporting this? where does the funds come from to coordinate and support those efforts? • How would a long-term medical monitoring of human patients be coordinated informatively? • Will the patients be expected to agree to lifelong follow up after treatment? How should this be achieved while preserving individual autonomy? • For each of the above questions, who should decide the answers to these questions? Based on what criteria?
**Ethical, legal, and social issues research (ELSI) of gene editing**	
Somatic cell gene Therapy	• Do we require any changes to the existing legal structure to tackle somatic gene therapy? If so, who would form the legal structure any further? • Are the principles and procedures present in clinical trials sufficient? • How can somatic gene therapy trials be performed and assessed? • Do we require specific patient protection or status in these trials? • What are the protocols to be established for patients undergoing these treatments (i.e.: consent, genetic counseling, follow-up monitoring)? • To what degree will commercial companies be willing, or be allowed to offer, potentially upon consumer request, treatments based on therapies, where so much vagueness regarding likely harm? • Which healthcare practitioners should engage in the implementation of somatic gene therapy and the care of patients receiving these treatments? • How are we going to ensure equal access to the technology? • How do we ensure the need drives the usage and not the technical imperative? • Who will determine on roles and obligations in this novel context? • What criteria will be used to select the eligible diseases/populations to be treated? • How do we ensure that research funding is distributed proportionally to the amount of gene editing work being carried out?
Germline gene therapy	• Will gene editing of human germ line cells, gametes and embryos be permitted in basic science, for better knowledge of human biology (i.e.: human development) and without planning to be used to establish modified human life? • Should gene editing of germ line cells, gametes, or embryos or any other cell resulting in heritable modification be allowed in a clinical setting for humans? • Would any principles or reasoning justify the use of germline gene editing in humans in a clinical context, given the existing ban on such techniques in many jurisdictions? • Why should we consider using germline gene editing in the clinic when there are alternative ways in which couples can have healthy (biologically related) children? Who will decide? Based on what criteria? • Before considering germline gene editing, would we first understand the risks and benefits of somatic gene editing? • What are the functions and duties of the various parties involved in those decisions? • How do commercial incentives and the technological imperative play a role in these decisions? • If we entertain gene editing for reproductive use, what criteria would be considered safe according to various stakeholders (scientist, ethicists, clinicians, policy makers, patients, lay public)? Who will set this safety threshold and based on what risk/benefit calculations? • If germline gene editing was allowed, how would the fact that for the first time, a human would be directly editing the nuclear DNA of another human in an inherited manner cause some form of segregation of types of humans? • If ever permitted, should germline gene editing be limited only for specific medical purposes with a particular high probability of developing a disease, and if so, does it matter if the risk is not 100%, but much lesser? • How do we define/demarcate medical reasons from enhancement? And, as was posed above for the use in somatic cells, for what medical conditions will gene editing be deemed suitable for use? What will the criteria be and who will decide?
**Stakeholder, engagement, education and dialogue (SEED) for gene editing**	
Planting SEEDs for gene editing	• What are the roles and obligations do various stakeholders have in developing and sustaining engagement, education and dialogue? • What will, and what should be the role of scientists and other academics in this type of popular media communications, and engagement activities? • As public engagement can have multiple goals, before each activity, we must consider: What are our objectives? And, what strategy of engagement will best meet these objectives? • How will the mass of voices we want to include in public engagement be weighed against each other? How are we to make sure every voice is heard? • What position will feedback and preferences of various stakeholders play in the discussion and decision-making process? How will those opinions be balanced and treated in policy making? • How can we ensure that public education is not limited to a token work package in science grants and/or to campaigns that try to persuade for or against gene editing? • How can we ensure that such public education and engagement is available to everyone, including in countries that currently may not have the resources to take on such SEED activities?

*Adapted and modified from Howard et al. (2018).*

## Modern Biotechnology in Malaysia

With the launching of the National Biotechnology Policy (NBP) by the former Prime Minister of Malaysia, Datuk Seri Abdullah Ahmad Badawi, in 2005, Malaysia expressed its intention to engage in the biotechnology arena on par with the advancement of the 21st century (Ahmad Badawi, 2005; Quah and Arujanan, 2005). Malaysia offers a conducive environment for biotechnology investors due to numerous favorable factors such as being rich with various flora and fauna that can be developed into natural and medicinal/therapeutic products, having skilled human resources with a trained pool of talent for the biotechnology industry, and having good infrastructure for research and development (R&D) with modern facilities and state-of-the-art equipment for biotechnology research.

The Malaysian NBP, through its nine thrusts, would provide a comprehensive roadmap that would accelerate growth in the biotechnology industry (Arujanan and Singaram, 2018). The nine thrusts and its aim include: (i) agriculture biotechnology development to enhance the value of the agricultural sector, (ii) healthcare biotechnology development to strengthen the discoveries of natural products, (iii) industrial biotechnology development for the advancement of bioprocessing and biomanufacturing technologies, (iv) R&D and technology acquisition to foster multidisciplinary teams in research and commercialization initiatives, (v) human capital development in line with market needs through special schemes, programs and training, (vi) financial infrastructure development to provide funding and incentives to academia, private sector and government-linked companies, (vii) legislative and regulatory framework development to enable continuous reviews of the country’s regulatory framework and procedures in line with global standards and best practices, (viii) strategic positioning to build brand recognition for Malaysian biotechnology products, and (ix) government commitment to establish a professional implementation agency to oversee the development of the biotechnology industry.

The Malaysian Biotechnology Corporation (currently known as Malaysian Bioeconomy Development Corporation Sdn. Bhd.) was founded to serve as a one-stop organization to facilitate the involvement of companies in the biotechnology industry, implement government policies and initiatives, encourage research and development as well as commercialization, and create a robust investor ecosystem (Quah and Arujanan, 2005; Quah, 2007; Arujanan and Singaram, 2018). Meanwhile, to stimulate bio-entrepreneurship, BioNexus special status was awarded to qualified foreign and Malaysian biotechnology companies that provided incentives, grants, and capacity building programs to assist growth. Moreover, to complement the NBP, the Bioeconomy Transformation Programme (BTP) was launched in 2012 to accelerate its bioeconomy development.

Despite such initiatives, Malaysia’s biotechnology innovation faced critical and challenging implications (Mokhtar and Mahalingam, 2010; Arujanan and Singaram, 2018). Some of the stumbling blocks in Malaysian biotechnology include an imbalance between talent development and market needs, primarily due to the lack of skilled human capital and industrial bases, insufficient funding for biotechnology R&D, project duplication, absence of collaboration between research institutes and universities, lack of commercialization from research output, political appointments for top positions at government agencies and research institutes, and pursuing university ranking (i.e., QS World University Rankings, Times World University Rankings) through publications that dilute industrial engagement. In such circumstances, Malaysia should adopt a sectoral industrial policy by which the state directs resources to targeted industries identified as crucial for their future competitiveness. Furthermore, the biotechnology industry requires mobilization and efficient utilization of scientific expertise through training, education, and collaboration to build a competent and competitive industry. Interestingly, as Malaysia is a collectivist society, the development, commercialization, and success of modern biotechnology are primarily linked to public acceptance.

### Debate 2: What Is the Public’s Acceptance of Various Applications of Modern Biotechnology in the Malaysian Context?

A series of studies were conducted in the Klang Valley region among several stakeholders on acceptance of biotechnology in Malaysia. The respondents comprised of both genders, aged 18 years and above, had various educational levels and diverse racial and religious beliefs. The preliminary studies among this group showed a high level of awareness among biotechnologists and policymakers as they were directly involved in R&D or policy matters (Amin et al., 2007a; Amin and Ibrahim, 2011). On the other hand, the NGOs, media, politicians, and the general public exhibited a moderate level of awareness due to the limited exposure to modern biotechnology issues. The knowledge level of Buddhists and Christians was significantly higher than Muslims. The difference in educational exposure and deeply rooted religious beliefs may have contributed to these findings.

Following that, a re-evaluation study revealed an increase in overall awareness level compared to the previous assessment (Amin et al., 2011b). Once again, Muslim scholars displayed the lowest level of awareness. This suggests the importance of instilling more knowledge as Islam is the major religion in the country, and their permissibility of various modern biotechnology applications is often needed. Taken together, the level of awareness and knowledge is considered moderate in Malaysia, which calls for more effort and dissemination of information.

Acceptance toward modern biotechnology is predicted mainly by several categories of perception (i.e., general promise and concern of biotechnology, technology optimism, nature/materialistic value, predisposition toward Science and Technology (S&T), attachment to religion and custom), and attitude (i.e., familiarity, moral concerns, risks, risk acceptance, benefits, and encouragement) (Amin et al., 2007b, 2011d). The factors affecting public attitude toward modern technology are shown in Table 3. To evaluate GM soybean’s risk/benefit in Malaysia, a study was undertaken to analyze the perception and attitude parameters (Amin et al., 2006). The study concluded that factors predicting genetically modified (GM) soybean encouragement were linked to perception about the benefits, acceptance of risk, and moral concern. Overall, if the application offered clear benefits to consumers and were of low moral concern, the application would be highly encouraged (i.e., most respondents considered GM palm oil which was modified to reduce its saturated fat content with no gene transfer, highly acceptable) (Amin et al., 2008).

**TABLE 3:** Factors related to public perceptions, understanding, acceptance and ethical principles of modern biotechnology.

**Factors**	**Explanation**
**Attitude dimensions***^*a*^*	
Perceived benefit	• Usefulness or benefit was found to be a prerequisite for support. • If the applications were perceived to have significant benefits such as in health care, the applications were supported despite having some risks. • If the application was perceived to have only modest benefits, it was not supported even though the risks were perceived to be minor.
Perceived risk	• Perceived risk is also a substantial variable of encouragement. • If the perception of risks related to biotechnology is sufficiently high, no amount of benefits is likely to make it acceptable.
Risk acceptance	• Modern technologies that benefits are always accompanied by risks which posed serious dilemmas for societies. • ‘Revealed preference’ approach is based on the assumption that by trial and error, society has arrived at an “essentially optimum” balance between the associated risks and benefits. • ‘Expressed preferences’ approach measure public attitudes towards the risks and benefits from many activities and use the concept risk adjustment factor to establish levels of acceptable risks.
Moral concerns	• Societal and individual risk perceptions are proportional to moral values. • Individual who is willing to accept some level of risk, if the product was considered worthy and was not morally objectionable. • Fall into two classes: intrinsic (the process of modern biotechnology is objectionable in itself) and extrinsic (possible risks of different application of biotechnology).
Familiarity	• Whether a product contains a risky substance, whether the risk is known to science, and whether a person has control over consuming a certain product. • Five characteristics correlated highly with each other which reflected familiarity: observability, knowledge (known to those exposed), immediacy consequences, familiarity (not new) and known to science.
Encouragement	• Support or acceptability of a biotechnology application.
**General attitudinal classes***^*a*^*	
Knowledge and awareness	• More knowledgeable makes people more considerate to genetic engineering. • Perception of risk is higher amongst those with greater objective knowledge, and those who have discussed biotechnology over recent months, but such perception is low amongst those with little knowledge. • Acceptance of biotechnology by the public may not be related to awareness at all, in which regardless of whether individuals were aware of biotechnology, respondents were able to make a judgment about how useful or risky it was. • Those with more education may be better able to assess both risks and benefits of biotechnology critically.
Engagement	• Greater scientific knowledge is moderately associated with support for science. • ‘Attentive public’ approach: combine responses to the questions on awareness and talking to others about the subject of biotechnology. • ‘Informed citizen’ approach: people who have minimally heard of biotechnology and have a vocabulary of biological terms and concepts that is adequate for reading the science section of a major paper.
General value orientations (worldviews)	• Risk perception is defined by the norms, value systems, and cultural mannerisms of societies. • Those who are more concerned about nature are less optimistic about biotechnology, while those who embrace materialistic values are more optimistic.
General promises and concerns	• The general promise includes a set of items reflecting the promise of biotechnology to improve the quality of life. • The general concerns referred to the general reservations or concerns about the possible consequences of biotechnology. • People firstly form attitudes towards the overall risk and usefulness of the technology, and then only infer from these general attitudes how risky or beneficial a particular application of the technology is.
Confidence in key actors	• People come to know about new scientific discoveries and technological developments from the mass media such as television, radio, newspapers and books. • People often judge risk according to their perception of its controlling agents: if these controlling agents have a track record of secrecy, or they dominate supposedly independent regulatory bodies and the public policy process, then people magnify the perceived risks. • Without confidence in key players such as scientists, regulators, people are likely to have excessive perceptions of risks, as the assurances provided by the experts that the risks are low or manageable are treated with uncertainty.
Attitude toward Science and Technology/technology optimism	• Technology optimism refers to what the public feel about current technologies, whether they will improve his/her way of life in the next 20 years. • Those who are optimistic about one technology tend to be confident towards others. • Attitude toward Science and Technology or the impact of technology was found to influence risk magnitude and benefit of technological hazards.
Societal values (nature versus materialist)	• Ecological attitudes (which comprised of an aggregate of attitude towards environmental issues, impact of technology and post-material values) have shown considerable influence on both perceived risk magnitude and risk acceptance of technological risks. • Enthusiasts of biotechnology were found to believe in free-market economic (materialist), while the rejectors were more concerned about nature and the environment.
Demographic factors	• Demographic characteristics such as age and gender must be included because some researchers have argued that the continuing process of scientific discovery leaves older people behind and because men and women are known to differ on several science-related and technology-related topics. • Education needs to be included because of its strong connections with knowing and learning. • Peoples’ occupation and religious belief are also enduring characteristics that shape many social and political opinions on a wide range of topics.
**Ethical dimension***^*b*^*	
Rights theory	• Always act so that you treat human beings as autonomous individuals, and not as a mere means to an end. • Right of an individual to make choices about their own life, and not to be subjected to the imposition of others.
Theories of justice	• The society has to operate with such principles of justice that cater to the well-being of the less fortunate members of the society.
Consequentialism and utilitarianism	• Consequentialism argues that one knows what the appropriate action is, not based on universal duty, but rather based on the outcomes of one’s actions. • Discussions those around risk and benefit whereby it is the consequences of the use of biotechnology that are seen as important, rather than any pre-existing understanding of one’s duty or the appropriateness of maintaining a given set of relationships.
Precautionary principle	• Given the unknown and unpredictable consequences and risks of biotechnology, opposers argue that regulatory policy should approach biotechnology from the stance of the precautionary principle. • With the precautionary principle as the default mode of regulation, the regulatory policy should evaluate biotechnology for its human health, animal health, environmental, social, economic, cultural, ethical, and reciprocal impacts.
Environmental ethics	• ‘Human-centered’ approach: the environment is valued for what it can provide for humans, and we protect it so that the resources will be there for our use and that of future generations. • ‘Ecocentric’ approach: the environment is valued not for what it can give us, but because it has intrinsic value, separate from any value that we may provide it.
Religion	• The spiritual division refers to religion or the belief of individual or people. • Acceptance and success of biotechnology will be based on the ideological beliefs and the cultural values adopted by individual human beings, who, in turn, will shape societal beliefs and values. • There are principles or guidelines on how we should live, and what is the right thing to do in most religions.

*^*a*^Adapted and modified from Amin et al. (2007).*

*^*b*^Adapted and modified from Amin (2009).*

As mentioned previously, public acceptance is crucial to driving modern biotechnology forward, and one of the strategies would be using the influential role of media to disseminate information to the lay public. A study was performed to analyze the coverage of biotechnology issues in four mainstream Malaysian newspapers (i.e., Utusan Malaysia, Berita Harian, New Straits Times, and The Star) and correlated it to the Malaysian public awareness (Amin et al., 2011e). There was limited coverage in the newspapers, as within the span of ten years (2001–2010), only 729 news items on biotechnology were retrieved. Among the four mainstream newspapers, biotechnology issues were mostly covered by Malay newspapers, with Utusan Malaysia having the highest number of articles. As these newspaper companies are government-owned, government policies, the success of a research project, and the commercialization of products that promoted economic development or improved the standard of living in Malaysia were portrayed positively. Notably, Malaysia’s media failed to provide any room for discussion and debate, substantially reducing public education in the subject matter. It is also important to point out that these newspapers only covered policies and their implementation, thus minimizing exposure to modern biotechnology’s real content. Likewise, another study was undertaken to analyze the coverage of ethical issues of biotechnology in the mentioned Malaysian newspapers (Amin et al., 2011f). From the study, it was discovered that government ministers served as the primary source of information. Malaysians were exposed to various biotechnology ethical issues, whereby applications such as human cloning (a baby girl named Eve) were painted in a negative light. From a religious context, Islamic law forbids human cloning, while stem cells for medical or research purposes are widely accepted.

In such circumstances, the ethical dimension of modern biotechnology in Malaysia needs an immediate assessment. A study revealed that there were seven factors related to ethical aspects (Amin et al., 2009, 2011c), including labeling, risks to human health, whether biotechnology threatens the natural order of things, monopoly of the field, patenting rights, human rights to modify living things, and confidence in regulation. When confronted with these aspects, Malaysians were unsure whether a human has the right to modify living things and whether modern biotechnology threatens the natural order. The technology was perceived as having moderate risks to human health, and the public was moderately concerned about the monopoly of the modern biotechnology market by companies in developed countries. The respondents also had moderate confidence in government regulations and expected the authorities to play a larger role in regulation and providing safety. The respondents expressed a high level of need for labeling products to indicate product safety and acknowledged patenting rights of scientists and industries. There is a greater need to set the direction and pace of development in such circumstances to prevent questionable or premature commercialization of biotechnology products.

Another research was undertaken to assess five ethical aspects (familiarity, perceived risk, denying benefits if it is not developed, religious and ethical acceptance) of GM rice, which contained a synthetic mouse gene to enrich vitamin C (Amin et al., 2011a). Shockingly, unfamiliarity was observed among policymakers, although they were responsible for regulating current biotechnology issues. There were concerns regarding the extinction of the original species, potential risks to health, and long-term harmful effects of consuming the rice. The respondents with tertiary education considered GM rice more acceptable from their religious viewpoints than those with a lower level of education. In summary, the Malaysian public was doubtful about the transfer of a synthetic animal gene to plants. There is a need for clear guidelines on the permissible status of gene transfer to guide the Malaysian biotechnology industries in such a scenario.

Overall, Malaysian stakeholders in the Klang Valley region were perceptive on modern biotechnology applications and products (Amin et al., 2011d). Malaysian policymakers were reasonably optimistic about the development of modern biotechnology in Malaysia. Biotechnology knowledge differed across religions, races, ages, and education levels, but not gender. In contrast, awareness levels differed across ages, education levels, and gender, but not across religions and races. Religious attachment played a significant influence on the public’s perception toward modern biotechnology applications, with the Malays being most positively influenced by religion, followed by Indians and Chinese. Finally, all biotechnology applications were moderately accepted by respondents from all races, ages, and educational backgrounds. Public perception, understanding, and awareness can influence commercial introduction and adoption of the new technologies. The acceptance of genetic modification in different areas of application was linked to attitude, which is influenced by socio-demographic variables, knowledge about genetics and biotechnology, and the perception of personal health risks.

Focusing on health biotechnology (HB) in Malaysia, there are numerous challenges to successful innovation (Abuduxike and Aljunid, 2017). Firstly, there is a lack of a conducive innovation system for sustainable HB due to insufficient expertise in universities, and limited communication between universities, research institutions, health biotech firms, and government agencies. Secondly, inadequate funding due to bureaucracy and lack of transparency in funding allocation, especially for commercialization and long-term R&D and HB product development. Thirdly, shortage of local human capital and a wrong mindset of new graduates, where the training curriculum does not cater to the practical skills needed in the industry. Fourthly, the research areas are extensive, unfocused, and do not reflect the strengths of Malaysia. Finally, there are too many government policies and regulations, such as lack of a clear framework, lack of an effective commercialization chain, trouble registering, patenting products locally, and poor implementation. In such instances, Malaysia must be proactive to improve the current situation before embarking on its journey toward developing a successful, innovative, and sustainable HB.

## Regulations and Guidelines in Malaysia

To further strengthen the efforts of NBP, the National Institutes of Biotechnology Malaysia (NIBM) was established to administer three national biotechnology institutes, namely the Malaysian Institute of Pharmaceuticals & Nutraceuticals (IPHARM), Agro-Biotechnology Institute Malaysia (ABI), and Malaysia Genome Institute (MGI). Biomedical product and clinical translation regulations such as human cell- and tissue-based products are governed by the Ministry of Health (MOH) (Idrus et al., 2015). Thus, the Medical Development Division of MOH formulated four standards, including the Guideline of Cell and Gene Therapy Products (CGTPs) to regulate all industrial players in the field.

### Guidelines for Stem Cell Research and Therapy (2009)

In Malaysia, stem cell research is developed mainly in MOH facilities and university hospitals (Ministry of Health Malaysia, 2012). MOH is actively involved in stem cell regulations and provides numerous frameworks to guide researchers, clinicians, and companies in research, clinical trials, and manufacturing. A Guidelines on Stem Cell Research and Therapy was established, which highlighted that (Ministry of Health Malaysia, 2009b): (i) all experiments and clinical trials must be driven by a solid foundation of essential scientific and animal experimentation and must adhere to the highest medical and ethical standards, (ii) research on human adult stem cells, non-human stem cells and embryonic stem cell lines are allowed, and (iii) research on stem cells derived from fetal tissues of legally performed termination of pregnancy is permitted. On the other hand, the following are not permitted under the guidelines: (i) an *in vitro* culture of any intact human embryo, development from the fusion of human stem cell or any pluripotent cells with non-human cells, for more than 14 days or until the formation of the primitive streak begins, (ii) the introduction of human embryonic stem cells (hESC) into non-human primate blastocysts or in which any embryonic stem cells (ESC) are introduced into human blastocysts, and (iii) breeding of animal into which hESC have been introduced at any developmental stage.

The guideline only considers interventions at the *in vitro* level, animal studies, or clinical trials to sufficiently show safety, quality, and efficacy. Nevertheless, the currently accepted clinical application of stem cell- or/and cell-based therapies such as bone marrow or peripheral blood stem cell transplantation are limited to leukemia, lymphomas, and certain malignancies. The implementation of other clinical cases, including heart failure, stroke, spinal cord injuries, and organ failures, is still experimental. Nevertheless, in 2016, a pilot clinical trial, led by a team of orthopedic surgeons and stem cell scientists from Universiti Kebangsaan Malaysia (UKM), succeeded in treating a group of patients for knee articular cartilage defects using unmatched donor umbilical cord-derived mesenchymal stem cells (Rahim, 2019).

### National Standards for Stem Cell Transplantation (2009)

Stem cell therapy showed promising medical intervention for the treatment of malignancies in Malaysia. For example, the survival rate improved significantly for acute leukemia, with more than 50% fully cured because of bone marrow transplants (Murugappan, 2019). Thus, MOH increased its efforts in framing standards and guidelines to keep up with this technology. The National Standards for Stem Cell Transplantation was published to cater to the collection, processing, storage, and infusion of hemopoietic stem cells (HSC) and other therapeutic cells (Ministry of Health Malaysia, 2009e). The standards aimed to ensure the safety and efficacy of the product to be infused into the recipient. At present, the rules allow minimal manipulation of the cells/tissues whereby: (i) the processing of structural tissue should not change the original relevant tissue’s characteristics through reconstruction, repair, or replacement, and (ii) the processing for cells or non-structural tissue should not alter related tissue’s biological properties. In such circumstances, the processes of cutting, grinding, shaping, centrifugation, soaking in antibiotic or antimicrobial solutions, sterilization, irradiation, cell separation/concentration/purification, filtering, lyophilization, freezing, cryopreservation, vitrification is considered minimal manipulation, and any other alteration is subjected to scientific consideration and would have to be evaluated by experts. Notably, specialized processing procedures such as gene manipulation and insertion of new genetic material are only allowed after approval from an institutional review board or human ethics committee.

### National Guidelines for Hemopoietic Stem Cell Therapy (2009)

The advancement of stem cell therapy drove Malaysia to set up the National Stem Cell Coordinating Centre, a database of all registered donors for peripheral blood, bone marrow, and umbilical cord blood (Aruna, 2014). Moreover, the National Guidelines for Hemopoietic Stem Cell Therapy was released by MOH to provide standards for any medical facility in performing hemopoietic stem cell transplantation (HSCT) (Ministry of Health Malaysia, 2009c). HSCT is routinely performed for patients with malignant and non-malignant hematological conditions, solid organ tumors, inherited metabolic, and primary immunodeficiency diseases. Moreover, experimental procedures must be performed as clinical trials, and ethics approval should be obtained and adhere to National Guidelines for Stem Cell Research and Therapy.

At this juncture, evidence-based outcomes from all stages of clinical trials are needed to ensure the intervention will be safe and effective (Fiona, 2016). In the future, health and regulatory bodies such as the Medical Research and Ethics Committee (MREC), Medical Service Development Division of the Health Ministry, Clinical Research Centre (CRC), Clinical Research Malaysia (CRM), National Pharmaceutical Regulatory Agency (NPRA), National Stem Cell Research and Ethics Sub-committee (NSCERT), Institute for Medical Research (IMR), Malaysian Stem Cell Registry, the various ethics committees at higher learning institutions and medical centers, the BioMedical Division of Biotech Corporation, investors, fund providers, and other stakeholders, can engage with the public to provide more awareness on the progress of cell therapy and the funding mechanisms involved in the clinical trials.

### National Standards for Cord Blood Banking and Transplantation (2009)

Cord blood banking is gaining popularity among Malaysian parents, especially with the emergence of many private cords blood banking facilities in local settings such as StemLife and CryoCord (Goh, 2013). By preserving and storing blood taken from a baby’s umbilical cord right after birth, these companies state that they can treat blood disorders, including thalassemia, leukemia, and bone marrow failures. Thus, The National Standards for Cord Blood Banking and Transplantation was developed to guide cord blood collection facilities to process, test, bank, select, release, and uphold quality medical and laboratory practices in cord blood banking (Ministry of Health Malaysia, 2009d).

### Checklist for Research on Stem Cell and Cell-Based Therapies (NSCERT 2009)

The National Stem Cell Research and Ethics Sub-committee (NSCERT) developed a standard checklist for any application related to research on stem cell and cell-based therapies (Ministry of Health Malaysia, 2009a). The following procedures should be followed during submission: (i) all applications from MOH and the private sector must be submitted to MREC and registered under National Medical Research Register (NMRR); meanwhile, applications from universities must obtain approval from respective Institutional Review Board (IRB) or Independent Ethics Committee (IEC), (ii) upon review, a complete application will be forwarded to NSCERT for recommendation, (iii) NSCERT will make recommendations based on the proposed scientific evidence, (iv) NSCERT’s recommendations will be submitted to MREC/IRB/IEC, and applicants will be informed about the final decision.

### Guidance Document and Guidelines for Registration of Cell and Gene Therapy Products (CGTPs) in Malaysia (2016)

In general, CGTPs are categorized for “treating or preventing diseases in human beings, or administered to human beings with a view of restoring, correcting or modifying physiological functions by exerting pharmacological, immunological or metabolic action” (Ministry of Health Malaysia, 2016). In such circumstances, they are classified as medicinal products under the Sale of Drugs Act 1952: Control of Drugs and Cosmetic Regulations 1984 [P.U.(A) 223/84] (Laws of Malaysia, 1984). Under Part III: Registration and Licensing, Clause 7 (1), “no person shall manufacture, sell, supply, import, possess or administer any products unless the product is a registered product, and the product holds the appropriate license required and issued under these regulations.” Moreover, due to the increase of CGTPs, the ministry divided the control and regulation into three approaches where: (i) the clinical use/medical procedure of the product will be under the ambit of Medical Development Division, and Medical Practice Division of the MOH, (ii) the device element of such products must comply with the Medical Device Act and regulations under the ambit of Medical Device Authority (MDA), and (iii) the National Pharmaceutical Control Bureau (NPCB) [currently known as National Pharmaceutical Regulatory Agency (NPRA)] will ensure the medicinal product’s quality, efficacy, and safety.

This guideline covers cell therapy, xenotransplantation, and gene therapy, predominantly focusing on human stem cells, human tissue therapy products (e.g., skin, cardiovascular, ocular, musculoskeletal tissues), human cellular therapy products (e.g., cartilage cells, pancreatic islet cells, cultured skin cells, hematopoietic stem/progenitor cells derived from peripheral and cord blood), genetically modified cellular products, cell-based cancer vaccines, cell-based immunotherapies, and dendritic cells, lymphocyte-based therapies, cell-based therapies for cancer, peptides, and proteins. For gene therapy, the products may include recombinant nucleic acid sequences of biological origin, genetically modified viruses, genetically modified microorganisms, and cells altered by one or more of these substances. These products are widely classified based on the delivery method, such as viral vectors, nucleic acids in a simple formulation (naked DNA), and nucleic acids formulated with agents such as liposomes. Furthermore, the regulation also outlines the quality of biotechnological products, starting materials used to manufacture the active substance, materials used in culture, and preservation of the cells. The development of CGTPs guidelines in Malaysia is crucial to increase safety and control, promote sound science and its practical application in cell therapy.

The risk of cell-based therapy must be assessed through stringent regulation and oversight, and currently, there are two classes of products that have been identified. Firstly, the lower risk cell therapy products must be minimally manipulated, intended for homologous use only as determined by labeling, does not involve combination with another drug/article/device, and does not have a systemic effect. The product is regulated by the Medical Practice Division, donor screening and testing, and Good Tissue Practices. Secondly, the higher risk cell therapy products are used for other than normal function, is combined with non-tissue components, or is used for metabolic purposes and regulated as a biologic product. The quality and scientific evaluation must be adequately addressed to evaluate the product’s effectiveness and safety.

### Debate 3: Are the Current Standards and Guidelines Sufficient to Govern Gene Editing?

The activities related to the stem cells are predominately guided by the documents discussed above, which suggests good practices and guidelines, and are not legally binding regulations. In the absence of such regulations, there are no legal consequences when a person violates the practices recommended in the instruction (Gopalan et al., 2019). Besides a lack of legal framework, there are also overlapping guidance documents (i.e., Guidelines for Stem Cell Research and Therapy, National Standards for Stem Cell Transplantation, National Guidelines for Hemopoietic Stem Cell Therapy), thereby causing confusion among researchers and clinicians. Even though MOH released a ‘Checklist for Research on Stem Cell and Cell-Based Therapies,’ the document fails to address the issue of non-compliance and accountability (Gopalan et al., 2017). As the guidelines are deemed adequate and updated, MOH decided against establishing any legal document specifically for stem cells (i.e., Stem Cell Act) to govern the activities (Ministry of Health Malaysia, 2012). However, the absence of regulatory policies or any legal documentation may enable exploitation to generate profit in stem cell research and technologies, with unknown consequences. Nevertheless, the former Deputy Health Minister, Dr. Lee Boon Chye, announced that the CGTPs guidelines would be enforced from 2021 to safeguard public health (Bernama, 2018; Chung, 2018).

Respondents in a survey compared the jurisdiction between the current Malaysian stem cell research to other national regulatory agencies such as the US FDA and the UK’s Human Fertilization and Embryology Authority (Abdul Aziz et al., 2018). They believed that active engagement with regulators was crucial to guide what can be done in research and therapy. The respondents felt that the existing Malaysian guidelines were variable and limited, and there was a disconnect between written regulations and the day-to-day encounter by the clinical laboratory and scientists. There were mixed responses regarding the current regulatory regimen, wherein some regarded the framework as overly restrictive and hindered research advancement. Simultaneously, some claimed it was excessively facilitative due to the lack of monitoring and enforcement. This tug-of-war between regulation and scientific development in trying to stay abreast with neighboring countries while preventing irresponsible experimentation is undoubtedly challenging. In such circumstances, inspection and regular personnel training would play an essential role in maintaining quality and reducing incidences (Idrus et al., 2015). Even though there are no reports of misconduct, fraud, or deaths involving stem cell research in Malaysia, one cannot rule out the possibilities (Abdul Aziz et al., 2018; Gopalan et al., 2019). Without any formal complaints, no action can be taken. At present, the regulatory policy contains numerous loopholes such as overlapping of contents and is non-legally binding. Therefore, the solution lies in improving current guidelines, including a practical legislation framework.

Although there are many dilemmas about stem cell research in Malaysia, it is unclear whether gene editing is captured under any standards and guidelines. Given the current international proposals, Malaysia could adopt some of the elements in formulating policies addressing gene editing while adding its own historical, economic, social, and cultural perspective. It was perceived that public consultation would be an alternative option to direct governance of research and clinical applications using human gene editing (Alta Charo, 2016). Moreover, voluntary self-regulation and/or self-imposed rules could potentially restrict aspects of tissue donation, donor recruitment, and experimental procedures. A notable example of voluntary self-regulation is the Asilomar 1975: International Congress on Recombinant DNA Molecules, which declared a voluntary moratorium on recombinant DNA experiments by reviewing its potential hazards before pushing it forward (Barinaga, 2000; Berg, 2008). The experts agreed that research should be continued, but with stringent restrictions that estimate recombinant DNA technology risks and formulated ways of minimizing them. At the time, even without legislative restrictions, this moratorium proved that research could be undertaken as some scientists could self-govern. Notably, the congress community comprised primarily of academicians who may not have had a financial conflict of interest. Since then, the scientific era has changed drastically, genetic engineering has gone commercial, and a number of academics have shifted to biotechnology companies. In such a scenario, self-moratorium may not be feasible as many would have to adhere to company policies and the profit margin.

Regulation and legislation are crucial to manage emerging technologies for the public’s benefit. For instance, Japan has a regulative pathway that classifies risks as high, medium, or low (Alta Charo, 2016; National Academies of Sciences Engineering and Medicine, 2017b). United States (US) regulates its medical devices similar to Japan; however, in drug products, the US treats them as equally dangerous and utilizes safety and efficacy rules. Likewise, Singapore follows a risk-based approach for cell therapy and determines whether the modifications are major or minor, homologous or non-homologous, and in combination with other products. On the other hand, Brazil established laws governing genetically engineered food, stem cell research, and cell therapy, including constitutional prohibitions on human tissue sale. Remarkably, the Biosafety Law in Brazil tackles gene editing issues, allowing somatic gene editing in human subjects. Ecuador’s constitution bans the use of genetic material for scientific research that violates human integrity. In Panama and Mexico, genetic modification for reasons other than severe disease treatment is punishable by a 2-to-6-year prison sentence. Similarly, Colombia also imposes a 1-to-5-year prison sentence for applications other than treatment, diagnosis, and research to alleviate suffering.

China has a formulated regulatory framework governing gene and cell therapy, and the State Food and Drug Administration plays a role in approving gene therapy products for commercialization. Additionally, legal guidelines for human embryo research and *in vitro* fertilization (IVF) procedures have been published by authorities of the People’s Republic of China (Ministry of Health China, 2001, 2003). At this point, it is worth visiting the issue of He Jiankui, who created gene-edited babies using the CRISPR/Cas9 system (Cohen and Normille, 2020; Dyer, 2020). Jiankui was sentenced to 3 years prison sentence and fined 3m yuan (£329 000; €386 000; $430 000) by the Chinese court for fabricating an ethics review certificate. Jiankui and his team were also convicted of practicing medicine without a license, deliberately violating national regulations in scientific research and medical treatment. This implies that China has no strict regulations specific to gene editing and calls for rules relating to the genome to be included in the civil code (Cyranoski, 2019a,b).

Comparing the regulatory framework to a Western context such as the US or European Union (EU) could serve as a potential model to strengthen regulations and legal policies for gene editing in Malaysia, as summarized in Table 4 (Grant, 2016; Samori and Rahman, 2016; Halioua-Haubold et al., 2017). In the US, the FDA controls numerous products ranging from food, tobacco, vaccines to therapeutics. Gene therapy products are strictly regulated under Section 351 of the Public Health Service Act (PHSA), which covers “virus, therapeutic serum, toxin, antitoxin, vaccine, blood, blood component or derivative, allergenic products, or analogous products, … applicable to the prevention, treatment, or cure of a disease of human beings.” European Medicines Agency (EMA) is the centralized regulatory authority in the EU. Gene therapy products are classified as Advanced Therapeutic Medicinal Products (ATMP) and are governed under the ATMP regulation that covers Gene Therapy Medicinal Products (GTMP), Somatic Cell Therapy Medicinal Products (CTMP), Tissue Engineered Products (TEP), and Combined ATMPs. The US FDA and the EU EMA released resources relevant to gene editing (Shim et al., 2017), as summarized in Table 5.

**TABLE 4:** Comparison between the gene therapy regulatory framework in United States (US), European Union (EU), and Japan.

	**Malaysia**	**US**	**EU**	**Japan**
Key regulatory structures	• Ministry of Health (MOH) • National Pharmaceutical Regulatory Agency (NPRA) • National Stem Cell Research and Ethics Sub-committee (NSCERT) • Medical Research and Ethics Committee (MREC) • Institutional Review Board (IRB) or Institutional Ethical Board (IEB)	• Food and Drug Administration (FDA) • Center for Biologics Evaluation and Research (CBER) • 351 Product • Office of Tissues and Advanced Therapies (OTAT)	• European Medicines Agency (EMA) • Committee for Medicinal Products for Human Use (CHMP) • Advanced Therapeutic Medicinal Products (ATMP) • Committee for Advanced Therapies (CAT)	• Pharmaceuticals and Medical Devices Agency (PMDA) • Center for Product Evaluation • Regenerative Medicine Product • Office of Cellular and Tissue-based Products
Name of product	Cell and Gene Therapy Products (CGTPs)	Gene Therapy Product	Gene Therapy Medicinal Product (GTMP)	Gene Therapy Product
Definition of gene therapy	• Contains an active substance which consists of a recombinant nucleic acid administered to human beings with a view to regulate, repair, replace, add or delete a genetic sequence. • Its therapeutic, prophylactic or diagnostic effect relates directly to the recombinant nucleic acid sequence it contains, or to the product of gene expression of this sequence.	• Mediate effects by transcription and/or translation of transferred genetic material and/or by integrating into the host genome and that are administered as nucleic acids, viruses, or genetically engineered microorganisms. • The products may be used to modify cells *in vivo* or transferred to cells *ex vivo* before being administrated to the recipient.	• Contains an active substance which consists of a recombinant nucleic acid administered to human beings to regulate, repair, replace, add or delete a genetic sequence. • Its therapeutic, prophylactic, or diagnostic effect relates directly to the product of genetic expression of this sequence.	• Articles which are intended to be used in the treatment of disease in humans or animals, and are transgened to express in human or animal cells.
Some main guidelines	• Guidance Document and Guidelines for Registration of Cell and Gene Therapy Products (CGTPS) in Malaysia (2016) • Checklist for Research on Stem Cell and Cell-based Therapies (NSCERT 2009)	• Long Term Follow-up After Administration of Human Gene Therapy Products; Guidance for Industry (2020) • Guidance for Industry: Preclinical Assessment of Investigational Cellular and Gene Therapy Products (2013) • Guidance for Industry: Guidance for Human Somatic Cell Therapy and Gene Therapy (1998)	• Guideline on quality, non-clinical and clinical requirements for investigational advanced therapy medicinal products in clinical trials (2019) • Quality, preclinical and clinical aspects of gene therapy medicinal products (2018) • Quality, non-clinical and clinical aspects of medicinal products containing genetically modified cells (2012)	• Regenerative Medicine Promotion Law (2013) • Act of Safety of Regenerative Medicine (2013) • Act on Pharmaceuticals and Medical Devices (2013)

**TABLE 5:** Relevant regulatory guidelines applicable for gene editing technologies adapted from the Food and Drug Administration (FDA), US and the European Medicines Agency (EMA), EU.

**Guidance titles**	**Year published**
**Food and Drug Administration (FDA), US***^*a*^*	
Manufacturing Considerations for Licensed and Investigational Cellular and Gene Therapy Products During COVID-19 Public Health Emergency; Guidance for Industry	2021
Human Gene Therapy for Neurodegenerative Diseases; Draft Guidance for Industry	2021
Interpreting Sameness of Gene Therapy Products Under the Orphan Drug Regulations; Draft Guidance for Industry	2020
Chemistry, Manufacturing, and Control (CMC) Information for Human Gene Therapy Investigational New Drug Applications (INDs); Guidance for Industry	2020
Long Term Follow-up After Administration of Human Gene Therapy Products; Guidance for Industry	2020
Testing of Retroviral Vector-Based Human Gene Therapy Products for Replication Competent Retrovirus During Product Manufacture and Patient Follow-up; Guidance for Industry	2020
Human Gene Therapy for Hemophilia; Guidance for Industry	2020
Human Gene Therapy for Rare Diseases; Guidance for Industry	2020
Human Gene Therapy for Retinal Disorders; Guidance for Industry	2020
Evaluation of Devices Used with Regenerative Medicine Advanced Therapies; Guidance for Industry	2019
Expedited Programs for Regenerative Medicine Therapies for Serious Conditions; Guidance for Industry	2019
Regulatory Considerations for Human Cells, Tissues, and Cellular and Tissue-Based Products: Minimal Manipulation and Homologous Use; Guidance for Industry and Food and Drug Administration Staff	2017
Same Surgical Procedure Exception under 21 CFR 1271.15(b): Questions and Answers Regarding the Scope of the Exception; Guidance for Industry	2017
Deviation Reporting for Human Cells, Tissues, and Cellular and Tissue-Based Products Regulated Solely Under Section 361 of the Public Health Service Act and 21 CFR Part 1271; Guidance for Industry	2017
Recommendations for Microbial Vectors Used for Gene Therapy; Guidance for Industry	2016
Design and Analysis of Shedding Studies for Virus or Bacteria-Based Gene Therapy and Oncolytic Products; Guidance for Industry	2015
Considerations for the Design of Early Phase Clinical Trials of Cellular and Gene Therapy Products; Guidance for Industry	2015
Determining the Need for and Content of Environmental Assessments for Gene Therapies, Vectored Vaccines, and Related Recombinant Viral or Microbial Products; Guidance for Industry	2015
Guidance for Industry: BLA for Minimally Manipulated, Unrelated Allogeneic Placental/Umbilical Cord Blood Intended for Hematopoietic and Immunologic Reconstitution in Patients with Disorders Affecting the Hematopoietic System	2014
IND Applications for Minimally Manipulated, Unrelated Allogeneic Placental/Umbilical Cord Blood Intended for Hematopoietic and Immunologic Reconstitution in Patients with Disorders Affecting the Hematopoietic System – Guidance for Industry and FDA Staff	2014
Guidance for Industry: Preclinical Assessment of Investigational Cellular and Gene Therapy Products	2013
Guidance for Industry: Preparation of IDEs and INDs for Products Intended to Repair or Replace Knee Cartilage	2011
Guidance for Industry: Clinical Considerations for Therapeutic Cancer Vaccines	2011
Guidance for Industry: Potency Tests for Cellular and Gene Therapy Products	2011
Guidance for Industry: Cellular Therapy for Cardiac Disease	2010
Guidance for Industry: Considerations for Allogeneic Pancreatic Islet Cell Products	2009
Guidance for FDA Reviewers and Sponsors: Content and Review of Chemistry, Manufacturing, and Control (CMC) Information for Human Somatic Cell Therapy Investigational New Drug Applications (INDs)	2008
Eligibility Determination for Donors of Human Cells, Tissues, and Cellular and Tissue-Based Products; Guidance for Industry	2007
Guidance for Industry: Guidance for Human Somatic Cell Therapy and Gene Therapy	1998
**European Medicines Agency (EMA), EU***^*b*^*	
Questions and answers on comparability considerations for advanced therapy medicinal products (ATMP)	2019
Guideline on quality, non-clinical and clinical requirements for investigational advanced therapy medicinal products in clinical trials	2019
Quality, preclinical and clinical aspects of gene therapy medicinal products	2018
Management of clinical risks deriving from insertional mutagenesis	2013
Risk-based approach according to Annex I, part IV of Directive 2001/83/EC applied to Advanced Therapy Medicinal Products	2013
Design modifications of gene therapy medicinal products during development	2012
Quality, non-clinical and clinical aspects of medicinal products containing genetically modified cells	2012
Creutzfeldt-Jakob disease and advanced therapy medicinal products	2011
Questions and answers on gene therapy	2010
Quality, non-clinical and clinical issues relating specifically to recombinant adeno-associated viral vectors	2010
ICH Considerations: oncolytic viruses	2009
ICH Considerations: general principles to address virus and vector shedding	2009
Follow-up of patients administered with gene therapy medicinal products	2009
Scientific requirements for the environmental risk assessment of gene-therapy medicinal products	2008
Non-clinical studies required before first clinical use of gene therapy medicinal products	2008
Guideline on safety and efficacy follow-up and risk management of advanced therapy medicinal products	2008
Non-clinical testing for inadvertent germline transmission of gene transfer vectors	2006
Development and manufacture of lentiviral vectors	2005

*^*a*^Adapted from FDA Cellular and Gene Therapy Guidances: https://www.fda.gov/vaccines-blood-biologics/biologics-guidances/cellular-gene-therapy-guidances.*

*^*b*^Adapted from EMA Multidisciplinary: gene therapy: https://www.ema.europa.eu/en/human-regulatory/research-development/scientific-guidelines/multidisciplinary/multidisciplinary-gene-therapy.*

Although Malaysia has made some progress in CRISPR technology, it requires more initiatives to strengthen its growth to be par with other developed countries (Hamid, 2018). Thus, it is the scientific community’s responsibility to engage with political leaders to further highlight the potential of gene editing (i.e., funding, law, and public engagement). The government needs to develop and implement a comprehensive national framework that guides genetic resources and biotechnology applications (Komen, 2012). International guidelines must be translated into federal laws and regulations, and a coordinated framework for biosafety should also be established. In such circumstances, governance on genetic products through gazetting the Biosafety Act 2007 is a practical effort in regulating the technology (Hafis Aliaziz and Ab Rahma, 2018).

## Biosafety and Biosecurity in Malaysia

A biosafety measure was drafted following acceptance of the Cartagena Protocol in 2003, led by the Ministry of Science, Technology, and Environment (Darsan Singh et al., 2019). In the following year, the ministry was reorganized as Ministry of Science, Technology and Innovation (MOSTI) and the Ministry of Natural Resources and Environment (currently known as the Ministry of Energy and Natural Resources). Since then, the Ministry of Energy and Natural Resources has taken the lead role in monitoring and enforcing the Biosafety Act 2007, under the regulation of four authorities, namely Department of Biosafety (DOB), National Biosafety Board (NBB), Genetic Modification Advisory Committee (GMAC), and Institutional Biosafety Committee (IBC) (Arujanan and Singaram, 2018).

### Biosafety Act (2007) and Biosafety (Approval and Notification) Regulation (2010)

The aim of Act 678: Biosafety Act 2007 is to “regulate the release, importation, exportation and contained use of living modified organisms (LMOs), and the release of products of such organisms, with the objectives of protecting human, plant and animal health, the environment and biological diversity” (Laws of Malaysia, 2007; Darsan Singh et al., 2019). Modern biotechnology (Part I, Section 3) is defined as “*in vitro* nucleic acid techniques, including recombinant deoxyribonucleic acid (DNA) and direct injection of the nucleic acid into cells or organelles, or fusion of cells beyond the taxonomic family, that overcome natural physiological reproductive or recombination barriers and that are not techniques used in traditional breeding and selection.” In this context, LMOs means “any living organism that possesses a novel combination of genetic material obtained through the use of modern biotechnology.” In Malaysia, the term LMOs and genetically modified organisms (GMOs) are used interchangeably. There are five categories (i.e., release, contained use, importation for release, importation for contained use, exportation) of activities involving LMOs regulated by the Act (Ministry of Natural Resources and Environment Malaysia, 2007).

The Act consists of seven parts (Laws of Malaysia, 2007; Zainol et al., 2011; Idris, 2013): (i) Part I touches on preliminary aspects such as citation, commencement, non-application, interpretation, and fees on activities that will be carried out, (ii) Part II covers the establishment and functions of NBB, GMAC, the appointment of Director General and other officers, (iii) Part III deals with release and importation activities which necessitate application for approval, (iv) Part IV discusses the notification of specific events of LMOs such as export, contained use and import, (v) Part V focuses on the risk assessment, risk management report and emergency response plan, (vi) Part VI and Part VII cater to the issue of enforcement, appeal, and other miscellaneous aspects.

The Biosafety (Approval and Notification) Regulation 2010 was released to cater to two major issues (Laws of Malaysia, 2010). Firstly, on the environment and human safety of LMOs and giving the public confidence in LMO products through the IBC that operates at the institutional level. The establishment of IBC is aimed to “provide guidance for safe use of modern biotechnology, to monitor activities dealing with modern biotechnology, establishing and monitoring the implementation of policies and procedures for the purpose of handling LMOs and determining the classes of Biosafety Levels for contained use activity for the purpose of modern biotechnology research and development undertaken within a facility.” Secondly, the regulation governs the approval, certification, and notification of any release and importation of LMOs and LMO products. Notably, the provision (Part VII, Section 25) includes socio-economic considerations such as “the changes in the existing social and economic patterns and means of livelihood of the communities that are likely to be affected by the introduction of the LMOs, and the effects to the religious, social, cultural and ethical values of communities arising from the use or release of the LMOs.”

### Biosafety Guidelines for Contained Use Activity of Living Modified Organism (2010)

It was reported that many protested against the application for a confined genetically modified (GM) rice field trial at the Malaysian Agricultural Research and Development Institute (MARDI) at Tambun Tulang, Perlis, claiming “genetic engineering is an inherently unpredictable process associated with unintended effects” (Goh, 2019; Sira, 2020). In reality, GM crops are evaluated using extremely stringent research protocols that ensure their safety (Arujanan, 2017). In such circumstances, a guideline to regulate the handling, storing, and transferring LMO without endangering humans, plants, animal health, the environment, and biological diversity was published.

In general, the Biosafety Guidelines for Contained Use Activity of LMOs divides the containment facility into five categories based on organisms, including genetic modification of microorganisms (GM-BSL), plants (GP-BSL), animals (GA-BSL), arthropods (GI-BSL), and aquatic organisms (GF-BSL). Under various containment facilities and levels (i.e., BSL-1, BSL-2, BSL-3, and BSL-4), a comprehensive description of the work practices, the minimum requirements for setting up facilities, and the required equipment under the different containment levels for contained use activities of LMO are provided. Moreover, the document also guides the disposal methods for biohazardous waste, as well as waste segregation and handling, whereby irresponsible disposal is prohibited and tightly governed by the Environmental Quality Act 1974, Environmental Quality (Scheduled Wastes) Regulations 1989, and Biosafety Act 2007.

A notification form must be submitted to IBC and NBB for any importation and exportation of LMOs. The LMOs must be clearly labeled and packaged in a tight container to avoid any material loss during transportation. The shipping of the LMOs starting from the research facility, storage facility, and field trial site should be recorded by IBC to ensure tracking. The LMO’s storage areas must be cleaned and clearly labeled, and access should only be permitted to trained authorized personnel. Furthermore, an inventory should be maintained to avoid unintentional release of LMO into the environment, and inspections should be recorded.

### Guidelines for Institutional Biosafety Committees (2010)

Institutional Biosafety Committee in any organization should be registered with the NBB and adhere to the Biosafety Act 2007 and Part II of the Biosafety (Approval and Notification) Regulations 2010. The Guidelines for Institutional Biosafety Committees: Use of LMOs and Related Materials was established to describe the setting up of the IBCs, its role, and scope, and processes that must be followed when obtaining, using, storing, transferring, or destroying LMO/recombinant DNA molecule (rDNA) (Ministry of Natural Resources and Environment Malaysia, 2010b).

Institutional Biosafety Committee plays a significant role to: (i) guide the principal investigator (PI) on biosafety policies for the use of LMO/rDNA research, the safety of laboratory personnel and other members of the organization, (ii) recommend and regularly review LMO/rDNA research that complies with Biosafety Act 2007 and Biosafety (Approval and Notification) Regulations 2010, (iii) monitor the facilities, procedures, practices, training and expertise of personnel involved in LMO/rDNA research, (iv) inform the PI of the results of the IBC’s review of all activities involving the use of LMO/rDNA, (v) evaluate and set containment levels for LMO/rDNA research, (vi) assess field experiments to make sure that the proposed risk assessment, risk management and emergency response plan are adequate, (vii) execute emergency response plan covering accidental spills and personnel contamination resulting from LMO/rDNA work, (viii) review and report to the head of the organization and to the NBB any notable problems with non-compliance of the Biosafety Act 2007 and Biosafety (Approval and Notification) Regulations 2010 and any significant research-related accidents or illnesses, and (ix) ensure that the information provided in the application form (Approval/Notification) is correct and complete.

In terms of modern biotechnology, the following activities must obtain IBC approval: (i) deliberate transfer of a drug resistance trait to microorganisms, (ii) intentional transfer of rDNA or DNA/RNA derived from rDNA into human research participants, (iii) deliberate formation of rDNA containing genes for the biosynthesis of toxin molecules lethal for vertebrates, (iv) use of Risk Group 2, Risk Group 3 or Risk Group 4 agents as host-vector systems, (v) cloning of DNA from Risk Group 2 or higher agents into non-pathogenic prokaryotes or lower eukaryotic host-vector systems, (vi) utilizing infectious or defective Risk Group 2 or higher agents, (vii) using whole animals in which the animal’s genome has been altered by the stable introduction of rDNA or DNA/RNA derived from rDNA into a germ-line, (viii) viable rDNA-modified microorganism tested on whole animals, (xi) genetically engineered plants by rDNA procedures, and (x) formation of rDNA material containing two-thirds or more of the genome of a eukaryotic virus.

### Malaysia Laboratory Biosafety and Biosecurity Policy and Guideline (2015)

In 2013, the Biosafety and Biosecurity Subcommittee of the National Technical Advisory Committee of Public Health Laboratory gathered local personnel’s input and expertise to implement effective biosafety practices and establish a Malaysia Laboratory Biosafety and Biosecurity Policy and Guideline. The document comprises basic concepts and approaches to regulate all activities involving handling, manipulation, working, using, storing, and disposing of infectious and potentially infectious agents/materials and microbial toxins in all laboratories in the country (Ministry of Health Malaysia, 2015). Furthermore, the guide is a useful reference for establishing good microbiological techniques (GMT), biosafety, and biosecurity in the laboratory and defined containment zones.

This document provides a comprehensive guide on basic administrative controls, engineering controls, standard operating procedures, and personal protection controls. In terms of administrative controls, the Institutional Biosafety and Biosecurity Committee (IBBC) is solely responsible for ensuring the policy and guidelines are implemented. The IBBC serves as the custodian for all the biosafety and biosecurity administrative controls for the organization. Meanwhile, the engineering personnel handles the physical containment facility (i.e., BSL-1, BSL-2, BSL-3, and BSL-4), infrastructure, design, safety, and security requirements.

Standard operating procedures (SOPs) are produced to ensure all routine laboratory activities and specific methods for handling particular microorganisms, pathogens, and toxins are reproducible when performed by any individual following the instruction. The IBBC establishes all SOPs related to infectious and potentially infectious agents/materials and microbial toxins. Besides that, personnel protective equipment (PPE) minimizes exposure to infectious agents and microbial toxins. PPE must be made available along with proper SOP. Laboratory biosafety checklist is also included in the document, covering three levels of containment (i.e., BSL-1, BSL-2, and BSL-3) facilities, including laboratory and its design, gas cylinders and chemicals handling/storage, refrigerators/freezers/cold rooms, electrical equipment, personal protective equipment, waste management, occupational health, and safety program, general engineering controls, general practices and procedures, general laboratory housekeeping, fire protection, biological safety cabinet (BSC), administrative controls, decontamination, handling of contaminated waste, and laboratory biosecurity.

### Draft Code of Conduct for Biosecurity in the Framework of Biological Weapons Convention (2015)

In considering the need for immediate action on biosecurity, a workshop was held in 2015 as a platform to discuss and present Malaysia’s draft of the National Code of Conduct for Biosecurity (Science and Technology Research Institute for Defence, 2015). This initiative aimed to create awareness on codes of conduct, define professional and ethical behavior, and come up with a mutual agreement on the code of conduct among the broader scientific community. Thus, a draft of code of conducts was established to raise awareness on potential dual-use and prevent malicious misuse, to assist research organizations avoiding any direct or indirect contributions to the development and production of potential biological weapons, to demonstrate that research organization are fully compliant with national and international legislation, and support the Biological and Toxin Weapons Convention (BTWC) as an international norm prohibiting biological weapons. The 10 significant elements of the draft are related to: (i) biorisk assessment and risk management, (ii) raising awareness, (iii) safety and security, (iv) education and information, (v) accountability and oversight, (vi) reporting misuse, (vii) internal and external communication, (viii) research and sharing knowledge, (ix) accessibility, and (x) supply, shipment and transport.

Biorisk assessment (BRA) and biorisk management (BRM) highlights the misuse of biological substances in hazardous applications either intentionally or due to a lack of risk assessment and management. It is crucial to restrict access of biological products to authorized personnel only, and the activities must be reviewed regularly by the organization in terms of resources, responsibilities, compliance, and communication for reliable BRA and BRM. All staff must be educated and regularly trained in dual-use aspects of biological products and biosecurity regulation, as well as be aware of the potential harm of product misuse. Scientists working with pathogenic organisms or dangerous toxins must adhere to safe and good laboratory practices. Moreover, scientists must take the initiative to disseminate information, convey national and international regulations, and establish policies to prevent the misuse of biological products.

Other than that, any scientist that becomes aware of activities that breach the BTWC or other international law must report the suspicion of the biological product, information, or technology directly to the appropriate authorities and agencies. Personnel involved in reporting would be protected from any unwanted consequences. In such a phenomenon, the scientist must fully observe principles and be responsible for overseeing research projects or publications. Access by unauthorized personnel to any internal and external data about potential dual use must undergo serious consideration. In terms of supply, shipment, and transport, all dual-use biological products should be screened by the relevant authorities and must be transported or exported carefully following applicable regulations. Implementing these elements of code of conduct for biosecurity will ensure safety and enable a secure environment to conduct responsible medical and life sciences work.

### Debate 4: Are the Current Biosafety and Biosecurity Guidelines Sufficient to Regulate Gene Editing?

The impact of biotechnology activities on environmental sustainability and biodiversity is a global biosafety concern. In such circumstances, the precautionary principle approach is crucial to ensure the safe use of GMOs. This principle seeks to predict the consequences of biotechnology and its application that may increase threats to human health or the environment and the precautionary actions that must be undertaken. Furthermore, the precautionary approach must also consider the bioethics principle in decision-making, as it is closely related to how technology may influence humans’ well-being, animals, and nature. In this case, a project on a field release of engineered mosquitoes [OX513A(My1)] into an uninhabited forested area of Bentong, Pahang, and Alor Gajah, Melaka was approved by NBB on 5 October 2010 [reference number NRE(S)609-2/1/3] (Lacroix et al., 2012). The application was approved based on recommendations by the GMAC and had successfully addressed concerns raised through public consultation (conducted for 30 days) (Ministry of Natural Resources and Environment Malaysia, 2010c; National Biosafety Board, 2010). Furthermore, information on the project was made available on the Biosafety Department website and published twice in a local newspaper (with a gap of 2 weeks).

Despite implementing a well-planned trial, some community groups were still dissatisfied with the public engagement process (Hamin and Idris, 2011; Idris et al., 2012, 2013; Subramaniam et al., 2012). It is uncertain whether the local communities in Bentong and Alor Gajah were included in the mandatory consultation before the board’s approval. Notably, individual informed consent was not obtained regarding the field trial as it was not feasible. Moreover, there was also a negative perception of the trial on the use of GMO technology. Indeed, the degree of communication explaining the risks and benefits of the field trial to public health was unclear. Revisiting the Biosafety Act (Part IV, Section 35), the word ‘may’ indicate that it is the discretionary power of the Board of Minister to consider socioeconomic values in evaluating GMOs. This provision conflicts with Part III, Section 15: “Advisory Committee shall assess such application for the purpose of making recommendations to the Board,” which is purely based on scientific evidence and not ethical ones. There is also vagueness in terms of public participation in decision making (Part VI, Section 60): “subject to the discretion of the Board, the public may have access to such information relating to any application for approval, approval granted or notification, which has not been granted confidentiality under subsection 59(2) in such manner as the Board thinks fit.” The word ‘manner’ could simply mean to preserve the commercial benefit if requested by the applicant. At this point, there is a lack of clarity on incorporating public and socio-economic considerations in the actual decision making. The Act seems overshadowed by diplomacy in accessing information by the public and the controlled manner related to its release.

In Denmark, the Danish Board of Technology encourages society’s active involvement in biosafety issues (Glover et al., 2003; Idris et al., 2012). In the United Kingdom, due to a lack of trust in science officials, it is crucial to provide as much information as possible to the public for biosafety approval. In Brazil, there is an attempt to broaden the public’s participation in biosafety evaluation, while in India, intensive media coverage and NGO demonstration have reflected a sense of insufficient engagement with the issue. There are collective attempts to engage civil society in developing the biosafety framework in Kenya and Zimbabwe despite constraints in resources and capacity. China has also sought to address biosafety issues within its governmental context, rather than a civil society where public participation has been widely incorporated into the decision-making. There are numerous existing international biosafety and biosecurity standards developed by World Health Organization (WHO) and Centers for Disease Control and Prevention (CDC) that can be applied to any institution globally (Bielecka and Mohammadi, 2014), as shown in Table 6.

**TABLE 6:** Relevant biosafety and biosecurity documents applicable for gene editing technologies adapted from the World Health Organization (WHO) and the Centers for Disease Control and Prevention (CDC), US.

**Guidance titles**	**Year published**
**World Health Organization (WHO)*^*a*^***	
Guidance on regulations for the transport of infectious substances 2019–2020	2019
Biosafety video series	2019
WHO Global Consultative Meeting on the Safe Shipment of Infectious Substances: 15–16 March 2018	2018
WHO consultative meeting high/maximum containment (biosafety level 4) laboratories networking: 13-15 December 2017	2018
Report of an extended meeting of the biosafety advisory group: 13–15 December 2016	2018
Extended Biosafety Advisory Group meeting, 24–26 November 2014	2015
Laboratory Biorisk Management: Strategic Framework for Action 2012–2016	2012
Responsible life sciences research for global health security	2010
Biorisk management: Laboratory biosecurity guidance	2006
Public health response to biological and chemical weapons: WHO guidance	2004
Laboratory biosafety manual: 3rd Edition	2004
**Centers for Disease Control and Prevention (CDC), US*^*b*^***	
Biosafety in Microbiological and Biomedical Laboratories (BMBL) 5th Edition	2009

*^*a*^Adapted from WHO Biorisk management: core documents: https://www.who.int/ihr/publications/bioriskmanagement_1/en/.*

*^*b*^Adapted from CDC Biosafety in Microbiological and Biomedical Laboratories (BMBL) 5th Edition: https://www.cdc.gov/labs/BMBL.html.*

It needs to be noted that it was not easy to get industries to accept the provision on “may also take into account socio-economic considerations,” which demands more transparency (Hamin and Idris, 2011; Ramatha and Andrew, 2012). In general, socioeconomic values can be considered during the development of a domestic biosafety regulatory regime, during the risk assessment for GMOs, after a risk assessment, and during the appeal, review, or renewal of a permit. The evaluations are based purely on the economic impacts such as the distribution of benefits, research and development efforts, social and cultural issues that include public opinion, and ethical considerations. It is indeed tricky, time-consuming, and cost-ineffective to have socioeconomic views in decision making.

Moving forward, the application of viral vectors for gene therapy plays a vital role in achieving therapeutic efficacy (Ghosh et al., 2020). Nevertheless, these methods pose a risk and are still being studied to safeguard safety and effectiveness. There are limited resources available at the national or institutional level in the Malaysian context to assess and minimize the risk of viral vectors in research or clinical areas. Viral vectors are permitted to be used in experiments provided that the DNA (Ministry of Natural Resources and Environment Malaysia, 2010a) introduced is fully characterized and will not increase the virulence of the host or vector, and does not comprise or represent more than two-thirds of the genome of a virus.

Biosafety caters to “containment principles, technologies, and practices that are implemented to prevent unintentional exposure to pathogens and toxins, or their accidental release,” while biosecurity refers to the “institutional and personal security measures designed to prevent the loss, theft, misuse, diversion or intentional release of pathogens and toxins,” and both require special attention (World Health Organization, 2018). It is widely acknowledged that Malaysia’s initiatives to promote modern biotechnology has encouraged the scientific community to explore genetic engineering. However, this may trigger the malicious use of technology for terrorist activities (Berns, 2014; Gronvall, 2014). The provision of the Biosafety Act 2007 and the Biosafety (Approval and Notification) 2010 regulations would have been inadequate to address biosecurity in Malaysia.

Considering those circumstances, Malaysian’s BTWC bill which was drafted in 2012 and Science and Technology Research Institute for Defence (STRIDE) under the Ministry of Defence (MINDEF) addressed the deliberate use of biological agents or toxins as a weapon (Science and Technology Research Institute for Defence, 2018). Nevertheless, ensuring compliance with the BTWC by all institutions in Malaysia, such as the personnel working with pathogens and toxins, engineered controls, and biocontainment facilities, remains a conflict (Subramaniam, 2014). The personnel must be qualified and well-trained to understand the biological agent’s containment conditions and how it can be safely manipulated and accessed. In such situations, the biorisk management committee (BMC) should be knowledgeable about biosafety and biosecurity legislation and its management. Currently, the appointment of a biosafety officer is predominately based on work experience and is responsible for implementing regulations in individual institutions or laboratories. Hence, the primary goal is to build a suitable ‘biorisk culture’ that comprises of proper biosafety and biosecurity practices and demonstrates responsible conduct at all levels in an organization.

Based on the discussion above, Malaysia’s biosafety law is somewhat ambiguous in addressing bioethical concerns (Idris et al., 2013). It is recommended that the acts find a balance between promoting the advancement of modern biotechnology, ensuring environmental and public health safety, and considering public engagement in decision-making. For effective engagement, the following may be practical (Quinlan et al., 2016): (i) employing a wide range of resources to promote public education on the latest technologies, (ii) defining the objectives before seeking input, (iii) interacting with public groups from which information is needed, (iv) employing a clearly defined approach in making biosafety decisions, and (v) avoiding technical jargon. Stakeholders, policymakers, and the research community must work closely to assess risks and benefits. The government should also take initiatives to regain public confidence to enable them to understand the regulations (Zainol et al., 2011). Specifically, Malaysian authorities should be diligent in addressing the misuse of genetic engineering as bioweapons/bioterrorism (Majid, 2012). The scientific community and policymakers must collaborate and take responsibility to prevent the accidental or deliberate release of biological agents.

## Conclusion and Future Directions

Emerging technologies being developed over the next 5–10 years would significantly impact the economy and society (Academy of Sciences Malaysia, 2017a,b). Notably, technologies such as gene editing will drastically change the way we think about healthcare and will likely eliminate hereditary diseases. Malaysia is expected to embark on various emerging science and technology areas, especially concerning the following initiatives: (i) genetic testing of inherited diseases, (ii) gene therapy, (iii) genetic profiling, (iv) gene editing, (v) gene-manipulated gamete, and (vi) gene therapy and stem cell treatment for degenerative diseases. Even though gene editing has much to offer, it is crucial to evaluate the implementation of this tool in medicine. In such a context, three giants influence the application of gene editing (Capps et al., 2017): (i) individuals involved in the development of the technology, (ii) institutions where research is housed, and applications transpire, and (iii) the prevailing cultures that exert influence in this area of study.

Firstly, individuals refer to researchers and policymakers, politicians, and administrators who create regulatory conditions in which gene editing occurs. The discovery is relevant if it translates into useful products; in this respect, the gene editing platform should be a public resource. Scientists and their institution depend on the public who volunteer their time, bodies and experiences for clinical trials through data and biosamples. The procedures use huge public time and resources, capital flow, and specific oversight and regulation. Thus, scientists are accountable to the public and should refocus their progress and investment in biomedical research based on public needs. Secondly, the contributing institutions, namely education, research and training, and security and stability, are the major research players. A broad partnership is necessary among institutions, researchers, participants, and the public. From this perspective, most public benefits from open science where researchers gather and share data, rather than conceal and withhold data. Thirdly, the value of trustworthiness is crucial, so the public expects their interest to be respected and considered in the pursuit of commercialization by the holding institution. Researchers may be hesitant about the concept of shared benefit and solidarity. Nonetheless, they should learn to prioritize the interest of the public without being critical of the process. Secrecy and hype-oriented misinformation may otherwise result in discouraging participation. Thus, by creating a safe space for engagement, information dissemination, honesty, and upholding research integrity, technology can be accelerated confidently.

At this juncture, it is essential to refocus gene editing laws and ethics toward clinical applications (Nicol et al., 2017). Notably, an overly rigid legislative response may prevent the researcher from undertaking gene editing work irrespective of the potential benefits. Mechanisms for periodic review need to be established to ensure responsiveness and re-evaluation of risks and benefits. Moreover, regulations must be adequately adopted to address new technological advances and applications as they arise. It is crucial to set different thresholds for acceptable risk-benefit ratios legitimately. By engaging a more comprehensive range of stakeholders (i.e., patients and families), the acceptable threshold for risks and benefits would be more apparent. Besides that, human research’s ethical evaluation may provide an opportunity for public participation, such as having diverse membership in the IBC.

Moving ahead, biosafety and bioethics are of vital concern. In Malaysia, there remains a substantial lack of awareness on the issue (Zainol et al., 2011). There is a need for greater exposure to biosafety concerns, and relevant information must be provided through appropriate education. Education also has to be conducted at all levels, including schools, universities, and the public (Rusly et al., 2011; Ndolo et al., 2018). For instance, classes on biosafety can be included as a minor subject at schools and universities. For the public, practical strategies can be utilized, such as workshops, seminars, forums, small discussion groups, and the dissemination of biosafety issues in the newspapers, radio, and television. The public needs to be informed of the facts to bring modern biotechnology forward (Idris et al., 2012; Ramatha and Andrew, 2012). These initiatives may explore the possibilities of including socio-economic aspects in decision making. Policymakers must also address the potential misuse of biological agents as bioweapons (Majid, 2012). Alongside GMAC reviews, STRIDE/MINDEF can provide input on whether genetic engineering research would potentially pose a danger to national security. Furthermore, to safeguard the application of modern biotechnology in Malaysia, it is vital to produce and train more experts in legal issues associated with gene editing.

Malaysia has many standards, guidelines, and policies to cater to modern biotechnology, as summarized in Table 7. Its future role can be enhanced by creating a balance between promoting the development of the biotechnology industry and ensuring environmental and public health safety. Based on our discussion, the outlook on gene editing is not transparent, and it is unclear whether the existing regime suffices to address the technology. Malaysia is still new to this technology and needs to address several areas before embarking on this modern technology in the current situation. In such a phenomenon, several international organizations have issued frameworks and guidelines that offer different approaches to safeguard the regulation, biosafety, and biosecurity aspects of gene editing. All responsible parties such as MOH personnel, policymakers, bioethics, legal researchers, and physicians should create a forum to discuss such recommendations to formulate a document for gene editing in Malaysia explicitly. The guidance must be authoritative and enforceable and should be up to international standards for human research.

**TABLE 7:** Summary of key ministry, regulatory bodies, and their publications to safeguard modern biotechnology in term of regulations and guidelines, biosafety and biosecurity in Malaysia.

	**Description**	**Objective(s) and focus**
**Regulations and guidelines**		
Key ministry	Ministry of Health (MOH)	• Facilitate and support the people to attain their potential fully in health, appreciate health as a valuable asset, take individual responsibility and positive action for their health. • Ensure a high-quality health system that is customer centered, equitable, affordable, efficient, technologically appropriate, environmentally adaptable, and innovative. • Emphasize professionalism, caring and teamwork value, respect for human dignity, and community participation.
Regulatory bodies	Medical Development Division	Develop medical services in the MOH’s hospital, in particular, the speciality and sub-speciality services.
	Medical Practice Division	Ensure safe, efficient and quality health care standards through monitoring, legislation, regulation and regulation.
	Medical Device Authority	Provide regulatory control of the medical device industry in Malaysia, through compliance of act by ensuring safety and performance to protect public towards excellent customer satisfaction.
	National Pharmaceutical Regulatory Agency (NPRA)	Safeguard the nation’s health through scientific excellence in the regulatory control of medicinal products and cosmetics.
Publications	Sale of Drugs Act 1952: Control of Drugs and Cosmetic Regulations (1984)	All drugs in pharmaceutical dosage forms and cosmetics must be registered before sales and marketing are permitted in the country.
	Guidelines for Stem Cell Research and Therapy (2009)	Facilitate researchers and clinicians from MOH, universities and private sector that are involved in stem cell research and therapy (adult stem cells and human embryonic stem cells)
	National Standards for Stem Cell Transplantation (2009)	• Laboratory framework to support stem cell therapy from the point of collection, processing, storage, handling and infusion of the products to ensure patients’ safety. • Standards apply to sources of cells currently used for transplantation and cell therapy (bone marrow, peripheral blood and umbilical/placental blood).
	National Guidelines for Hemopoietic Stem Cell Therapy (2009)	Standards for any clinical facility in Malaysia performing hemopoietic stem cell transplants.
	National Standards for Cord Blood Banking and Transplantation (2009)	Standards on cord blood banking for transplantation in both private and public cord blood banks in Malaysia
	Checklist for Research on Stem Cell and Cell-based Therapies (2009)	Describes some of the procedures to be followed in making applications for stem cell and cell-based research involving human subjects, prepared by National Stem Cell Research and Ethics Sub-committee (NSCERT).
	Guidance Document and Guidelines for Registration of Cell and Gene Therapy Products (CGTPs) in Malaysia (2016)	• Outline the concept and basic principles of CGTPs. • Introduce the registration framework and guidelines to be applied. • Provide applicants with a “user guide” for the relevant scientific data and information, to substantiate the claimed quality, safety and efficacy of the product.
**Biosafety and biosecurity**		
Key ministry	Ministry of Energy and Natural Resources	Provide exceptional services in the management of natural resources and conservation of the environment in line with the national vision.
	Ministry of Health (MOH)	*same as above*
	Ministry of Defence (MINDEF)	Protect and defend the national interest which is the cornerstone of the sovereignty, territorial integrity and economic prosperity of the nation
Regulatory bodies	National Biosafety Board (NBB)	The regulatory body for making a decision pertaining to the release, importation, exportation and contained use of any living modified organism (LMOs) derived from modern biotechnology.
	Department of Biosafety (DOB)	• Evaluate the applications for the release, importation, exportation and contained use of living modified organism, and the release of products of such organisms. • Carry out the monitoring and enforcement activities under the Biosafety Act 2007 • Provide technical advisory on the handling of living modified organisms. • Raise public awareness regarding the role of biosafety in human, plant and animal health, the environment and biological diversity. • Promote research, development, educational and training activities relating to biosafety.
	Genetic Modification Advisory Committee (GMAC)	• Makes decisions on LMOs use in Malaysia and to provide scientific, technical and other relevant advice to the NBB. • Identification and safety management of risks associated with the use of genetically modified organisms (GMOs) and products containing or consisting of GMOs.
	Institutional Biosafety Committee (IBC)	Monitor any work which involves the use of any LMOs, or recombinant DNA (rDNA) molecule materials conducted at or sponsored by the organization, irrespective of the source of funding.
Publications	Biosafety Act (2007)	Regulate the release, importation, exportation and contained use of LMOs, and the release of products of such organisms, with the objectives of protecting human, plant and animal health, the environment and biological diversity.
	Biosafety (Approval and Notification) Regulation (2010)	• Ensuring the environmental and human safety of LMOs and giving the public confidence in LMO products by established the IBC. • The activities covered include the approval, certification and notification of any release and importation of LMOs and LMO products.
	Biosafety Guidelines for Contained Use Activity of Living Modified Organism (2010)	• Identify the Biosafety Levels (BSL) for containment of any LMO activity. • Describe work practices under the various containment levels. • Outline the minimum requirements for setting up facilities for contained use activities of LMO. • Identify equipment requirements under the different containment levels.
	Guidelines for Institutional Biosafety Committees (2010)	Describes the setting up of the IBCs, its role and functions and also processes that must be followed when obtaining, using, storing, transferring, or destroying LMO/rDNA materials.
	Malaysia Laboratory Biosafety and Biosecurity Policy and Guideline (2015)	• Basic concepts and approaches in the form of policy and guidelines that govern all activities involving the handling, manipulation working, using, storing and disposing of infectious and potentially infectious agents/materials and microbial toxins in all forms and sizes of laboratories in Malaysia. • Reference for the development and establishment of the respective institutional code of practice for good microbiological technique (GMT), biosafety and biosecurity in a laboratory and defined containment zone.
	Draft Code of Conduct for Biosecurity in the Framework of Biological Weapons	• Raise awareness of potential dual-use and the need to prevent malicious misuse. • Help research institutions to avoid any direct or indirect contributions to the development and production of potential biological weapons. • Demonstrate that research institutions in the country are fully compliant with national and international legislation and support the Biological and Toxin Weapons Convention Nucleus (BTWC) as an international norm prohibiting biological weapons.

## Author Contributions

VK contributed to the conception and wrote the manuscript. KT participated in drafting the review and revising it critically for important intellectual content. All the authors reviewed the final manuscript to be submitted.

## Conflict of Interest

The authors declare that the research was conducted in the absence of any commercial or financial relationships that could be construed as a potential conflict of interest.
